# 

**Published:** 1811-02

**Authors:** 


### MONTHLY CATALOGUE OF NEW MEDICAL PUBLICATIONS.

Osteologia, or an Anatomical Description of the Human Bones: illustrated by fourteen accurate engravings, designed for the use of students. 12mo. Cox.

Anatomical Plates of the arteries of the Human Body, accurately coloured, and reduced from the Icones of Haller; with concise Explanations. Second Edition. 8vo. Cox.

Observations on the Act for Regulating Mad-Houses, and a correction of the statements of the case of Benjamin Elliott convicted of illegally confining Mary Daintree; with remarks addressed to the friends of Insane Persons. By James Parkinson. 8vo. Sherwood and Co.

Additional Cases with further directions to the Faculty, relating to the use of the Humulus Lupulus, or Hop, in Gout and Rheumatic Affections. By A. Freake. 8vo. Highley.

### NOTICES TO CORRESPONDENTS.

We are indebted to an intelligent writer who signs himself Senex, for some wholesome advice. The case, however, which called forth his animadversions, in our apprehensions hardly deserved them. He observes respecting it---" I have never visited America ; yet I would not give credit to any person, with whom I was not well acquainted, however *respectable,* who should tell me of *opening arteries in wrists and ankles.* I could never hear it without suspecting that he intended to impose upon my understanding ; and I shall never read it without hoping it to be an oversight of the writer or printer."

We assure Senex that in this instance there was no mistake of the printer, and we cannot for a moment suppose that any medical gen-man, much less Dr. Harrison, would attempt to deceive us with a false relation. Whatever opinion may be entertained of the propriety of taking blood from the arteries, at the wrists or ankles, the operation is practicable. Senex will find his wish for a case in favour of the Eau Medicinale d'Husson, gratified in our present Number. We shall be happy to receive the Case of " Change of Colour in a Lady," resembling that related by Mr. Goodwin in a recent Number.

We have been favoured with Communications from Dr. Bradley of Huddersfield, Mr. Baxter, Mr. Eagland, and Mr. Harrold.

The Commnnication from A. B. which has been accidentally overlooked, will appear in our next Journal, with the extract from the Repertory, with which A. B. wishes it to be ccompanied.

*Printed by E. Hemsted, Great New Street, Fetter Lane.*

